# Transmission Risk on a Neonatal Intensive Care Unit: *Escherichia coli* versus *Klebsiella pneumoniae*

**DOI:** 10.1155/2018/1525072

**Published:** 2018-04-29

**Authors:** Tanja Artelt, Martin Kaase, Ivonne Bley, Helmut Eiffert, Alexander Mellmann, Helmut Küster, Martina Lange, Simone Scheithauer

**Affiliations:** ^1^Infection Control and Infectious Diseases, University Medicine Goettingen, University Hospital Goettingen, Göttingen, Germany; ^2^Institute of Medical Microbiology, University Medicine Goettingen, University Hospital Goettingen, Göttingen, Germany; ^3^Institute of Hygiene, University Medicine Münster, University Hospital Münster, Münster, Germany; ^4^Department of Pediatric Cardiology and Intensive Care Medicine, University Medicine Goettingen, University Hospital Goettingen, Göttingen, Germany

## Abstract

Isolation precautions required for neonatal intensive care units are part of a bundle with the aim to prevent transmission, colonization, and infection with multidrug-resistant gram-negative pathogens as neonates face an increased risk of mortality and morbidity in case of infection. The following short report describes a transmission of 3MDRGN *Klebsiella pneumoniae* on a neonatal intensive care unit in a university hospital in Germany. This transmission occurred even though intensified infection control measures were in place, which impressively shows the importance of surveillance, outbreak management, and awareness of contributing factors regarding outbreak situations.

## 1. Introduction

Detection of multidrug-resistant gram-negative pathogens (MDRGN) in prematurely and maturely born infants with the need of intensive medical care causes major consequences. In case of infection, broad spectrum antibiotics are needed for treatment. Infections due to MDRGN are associated with worse outcomes compared to infections due to susceptible isolates. Isolation precautions required for neonatal intensive care units (NICU) are part of a bundle with the aim to prevent transmission, colonization, and infection with MDRGN [1, 2]. Due to the high impact of infections in the neonatal patient population, advice for interventions aiming at reducing transmission risks are more extensive than in the general patient population in some national recommendations [2–4]. According to international standards, single-room isolation is strongly advised for carbapenemase-producing *Enterobacteriaceae*. German standards go a step further and additionally recommend the isolation of patients with extended spectrum beta-lactamases- (ESBL-) producing *Enterobacteriaceae* in the neonatal ICU. Recommendations of the European Society of Clinical Microbiology and Infectious Diseases (ESCMID) for the general patient population are stratified according to species. They advise for single-room allocation only for *Klebsiella* ssp. harbouring ESBL genes but not for *Escherichia coli* containing the genes. This difference is based on the presumed different transmission potential on the species level. However, evidence is only limited and indirect.

In Germany, the following recommendations are given for neonatal ICUs (Table 1).

3MDRGN is defined as a gram-negative pathogen resistant to three of the following four different classes of bactericidal antibiotics in vitro: broad spectrum penicillins, third or fourth generation cephalosporines, carbapenems (in neonates meropenem), and fluoroquinolones. 4MDRGN is a pathogen with in vitro resistance to all of the abovementioned antibiotics. As fluoroquinolones are not empirically used in neonates, the definition of 2MDRGN, a gram-negative pathogen resistant to cephalosporines and broad-spectrum penicillins, is particularly important for neonatal ICUs.

In this context, current recommendations of the German Commission on Hospital Hygiene and Infection Prevention (KRINKO) at the Robert Koch Institute (RKI), Berlin, focus on a weekly screening for MDRGN [2, 3]. The global prevalence of colonization with MDRGN differs considerably. Considering the high influx of newly arrived refugees in 2015/16 mainly from high prevalence regions [5, 6], an advanced infection control strategy at the University Medicine Goettingen (UMG) was established. In addition to the implemented RKI recommendations, recently arrived pediatric refugees were isolated in single rooms immediately at admission and directly screened for MDRGN. For inpatient infants with a previous stay in abroad, screening for MRSA, 2MDRGN, 3MDRGN, 4MDRGN, and vancomycin-resistant enterococci (VRE) was established on the combined NICU at admission with the aim to reduce or even prevent transmissions [7].

## 2. Materials and Methods

### 2.1. Epidemiological Investigation

The combined neonatal-pediatric 20-bed ICU has the resources to care for ten extremely premature infants and ten pediatric patients with cardiac diseases.

An eight months old female refugee, who had recently arrived from Iran travelling via Turkey and Greece, was admitted to our combined neonatal-pediatric ICU in November 2015. The infant suffered from a severe cardiac malformation, but previous contact to the healthcare system had been denied several times. The patient was isolated and screened for resistant bacteria at admission in accordance with the internal UMG guidelines. 3MDRGN *Klebsiella pneumoniae* and 3MDRGN *Escherichia coli* were detected exclusively in a rectal swab without clinical symptoms. Therefore, the infant remained in single-room isolation (2015-11-19 to 2016-01-09). Being in a difficult general condition, the colonization evolved to an infection, and the infant needed specific antibiotic treatment.

**Figure 1:**
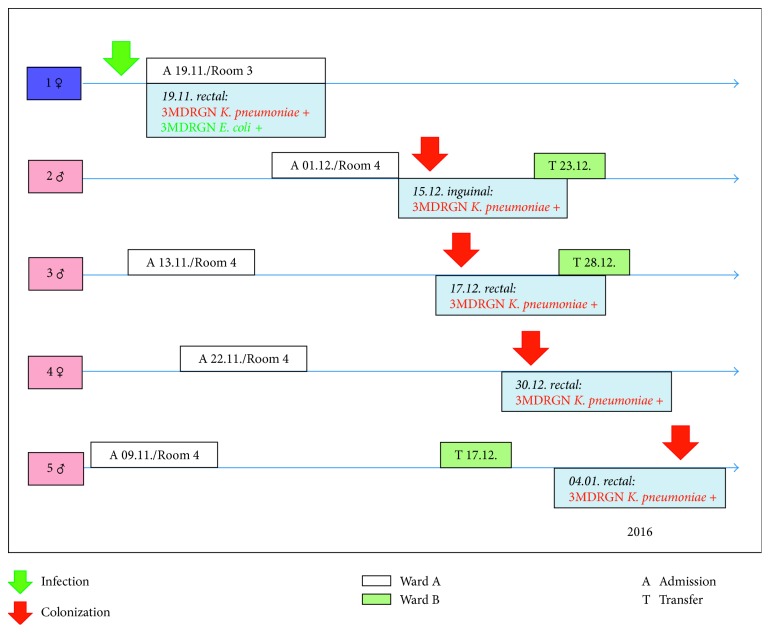
Transmission of 3MDRGN *Klebsiella pneumoniae* (linelist).

After a period of four weeks without any findings, we detected a colonization of *K. pneumoniae* in another infant with an identical antibiotic susceptibility profile within the weekly neonatal screening, but no *E. coli*. The infant shared an adjacent room with three more patients (Figure 1).

Consequently, a number of additional infection control measures according to the in-house standard operating procedure were implemented, as for cleaning twice daily not only the affected rooms, but the entire unit. Environmental screening included swabs from medical equipment and devices (ultrasound, X-ray, electrocardiography, and laminar air flow), swabs from surroundings in every patient room (e.g., disinfectant dispensers, soap dispensers, filter, and accessories for nursing including medical preparations like salves), and surroundings in parent rooms, kitchen, and laboratory. Extensive contact precautions for all patients on the ward, consequent isolation and cohorting of the four colonized patients, and all direct contacts were determined. Common areas as for the nursing and parent rooms were shut down, and conversations with parents and relatives in detail were accomplished. Furthermore, interdisciplinary rounds on the ward were arranged daily and documented in detail. Members of the executive board also were involved. All procedures were maintained until the end of January 2016.

### 2.2. Microbiological Methods

The swabs of the environmental screening were cultured in Caso-Bouillon (37°C/24 hours) and subcultured on nutrient agar plates, followed by differentiation and identification regarding morphological and biochemical characteristics.

The patient screening samples of the weekly screening for MDRGN were examined regarding the minimum inhibitory concentration (MIC) as part of determination of resistance via Vitek-MIC.

Two of five 3MDRGN *Klebsiella pneumoniae* isolates were recovered: patients number 2 and 3 (2015-12-15 inguinal swab and 2015-12-17 rectal swab, Figure 1). Whole genome sequencing (WGS) of the detected *Klebsiella pneumoniae* strains was performed on an Illumina MiSeq platform after DNA isolation and library preparation as previously described. Quality trimming, de novo assembly, and further analyses including core genome multilocus sequence typing were performed using SeqSphere+ software (Ridom, Münster, Germany) [8].

## 3. Results and Discussion

Despite preemptive isolation and infection control measures and according to advanced infection control strategies, a transmission of 3MDRGN *Klebsiella pneumoniae* occurred after a period of four weeks.

All environmental samples turned out to be negative for facultative pathogens except the swabs taken from the nearest surrounding in the index patient room. On devices like stethoscope, tape measure, several buttons, or xylocaine gel, only *Staphylococcus epidermidis* and aerobic spores could be detected. Many samples even turned out to be sterile. Microbiological results concerning medical devices and equipment in other rooms (patients, parents, kitchen, and laboratory) also turned out to be sterile or colonized with pathogens without relevance concerning infection control. The frequent exploration of inanimate surfaces detected *Enterococcus faecalis* on one thermometer. 3MDRGN *Klebsiella pneumoniae* or 3MDRGN *Escherichia coli* was not found, neither in the bedroom cohorting the four patients colonized nor in adjacent rooms. A point source could not be identified by investigating the samples. None of all patients developed an infection with 3MDRGN *Klebsiella pneumoniae* except the index patient.

The microbiological results of the weekly screening for MDRGN (rectal swabs) indicated the similarity of the 3MDRGN *Klebsiella pneumoniae* isolates (Table 2).

All five patient samples turned out to be positive for ESBL (Vitec-MIC). Development of gentamicin-resistance often appears quickly due to the fact that ampicillin/gentamicin is a first-line antibiotic in pediatric therapy.

**Figure 2:**
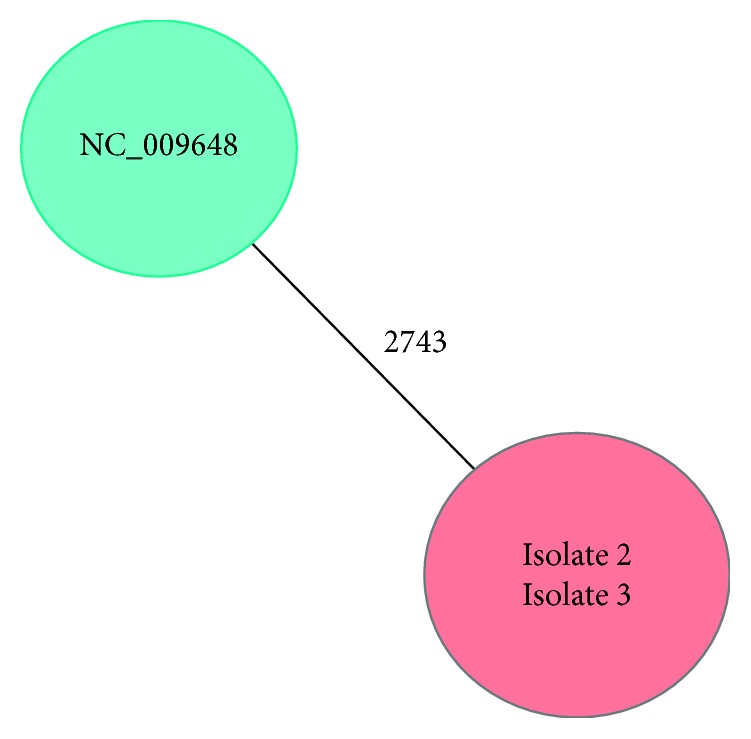
Minimum spanning tree analysis according to core genome MLST via SeqSphere+ based on reference genome (NC) and next generation sequencing (NGS).

All 3MDRGN *Klebsiella pneumoniae* isolates available (Isolate 2 and 3, corresponding to patient 2 and 3, Figure 1) were typed by NGS, and they turned out to be identical (Figure 2). The primary goal was to determine the clonal relationship of the isolates for infection control purposes. In this context, therefore it was only checked for the presence of extended-spectrum beta-lactamase genes. It was possible to extract the gene encoding CTX-M15 extended-spectrum beta-lactamase.

The retrospective analysis revealed an emergency situation that had happened on the NICU affecting the two patient rooms only a few days before detection of the 3MDRGN colonization—the same nursing staff had been involved in both of the rooms. Our hypothesis was a singular transmission during this situation.

Further consequences of the outbreak management were the closure of beds and the postponement of elective admissions on the combined neonatal-pediatric ICU as well as on the adjacent neonatal standard care unit.

Advanced infection control strategies were kept until the end of January 2016. Altogether 199 bed days at the neonatal-pediatric ICU and 122 bed days at the neonatal standard care unit were lost.

## 4. Conclusions

Despite the implementation of intensified comprehensive infection control measures, beyond official requirements, transmissions of MDRGN cannot be completely avoided in clinical settings. In this case, it is remarkable that a transmission of 3MDRGN *K. pneumoniae* but not of 3MDRGN *E. coli* occurred. This is in accordance with recent findings that *K. pneumoniae* is more transmissible than *E. coli* [9–11].

It becomes clear that proactive hygiene interventions influence the daily routine care on the NICU permanently. These interventions include strict isolation regimes for patients with colonization or infection as well as patients with contact to the index patients. Nevertheless, it is essential to remember basic infection control measures and to observe compliance of hand hygiene, contact precautions, and take care of staff training and instruction [12].

Pediatric refugee patients have a considerably higher prevalence of colonization with MRSA as well as cefotaxime and/or ceftazidime-resistant *E. coli* and *K. pneumoniae* than nonrefugee pediatric patients, as seen in the described case and in the data analyzed at our university hospital [7]. Screening for multidrug-resistant bacteria is considered to be of importance for infection control purposes and may offer an additional benefit for the empiric antibiotic treatment of infections in this population. Therefore, screening of pediatric refugee patients will be continued at our institution [5, 7, 13, 14].

## Figures and Tables

**Table 1:** Isolation precautions required for NICU [1, 2].

Pathogen	Single room	Cohorting	Protective gowns and disposable gloves	Mask
2MDRGN	−	+	+	−⁺
3MDRGN	+	+	+	+
4MDRGN	+	+	+	+

⁺Only at treatments with increased risk (e.g., ventilation procedures).

**Table 2:** Antibiotic susceptibility, 3MDRGN *Klebsiella pneumoniae*, and rectal swabs.

Antibiotic	Patient 1 (index)	Patient 2	Patient 3	Patient 4	Patient 5
Ampicillin	R	R	R	R	R
Ampicillin + Sulbactam	R	R	R	R	R
Piperacillin + Tazobactam	R	R	R	R	R
Cefuroxim	R	R	R	R	R
Cefotaxim	R	R	R	R	R
Ceftriaxon	R	R	R	R	R
Ceftazidim	R	R	R	R	R
Imipenem	S	S	S	S	S
Ertapenem	S	S	S	S	n.d.
Meropenem	S	S	S	S	S
Gentamicin	S	S	R	R	R
Cotrimoxazol	R	R	R	R	R
Moxifloxacin	R	R	R	R	R

R = resistant; S = sensible; n.d. = not done.
